# Acupuncture and Herbal Medicine for Cancer Patients 2014

**DOI:** 10.1155/2014/326179

**Published:** 2014-08-28

**Authors:** Thomas Efferth, Sookyung Lee, Yoshiharu Motoo, Sven Schröder

**Affiliations:** ^1^Department of Pharmaceutical Biology, Institute of Pharmacy and Biochemistry, Johannes Gutenberg University, Staudinger Weg 5, 55128 Mainz, Germany; ^2^Department of Clinical Oncology, College of Korean Medicine, Kyung Hee University, 149 Sangil-dong, Gangdong-gu, Seoul 134-727, Republic of Korea; ^3^Department of Medical Oncology, Kanazawa Medical University, Uchinada, Ishikawa 920-0293, Japan; ^4^HanseMerkur Center for Traditional Chinese Medicine, University Medical Center Hamburg-Eppendorf, House O55, University Clinic Campus, Martinistraße 52, 22303 Hamburg, Germany

Traditional medicine is a popular option for patients to get medical information and care with various reasons worldwide. Developing countries depend on it because of the affordable prices, while a large portion of patients of developed or industrialized countries have a continuing interest induced by the reluctance towards chemical drugs and mechanical approaches to disease. CAM offers a multitude of advantages for patients, especially in the chronic and life-threatening severe ailments such as cancer. The global demand for CAM makes it mandatory to investigate its scientific evidence basis.

Among the different facets of CAM, acupuncture and herbal medicine raised special interest not only for treatment of patients but also for research of medicine. As can be seen in Figure 1, acupuncture and herbal medicines have become thriving fields of research during the past half century. A comprehensive survey of published papers from 1960 to 2013 shows the number of annually published papers dealing with acupuncture or herbal medicines for all medical fields (Figure 1(a)) and in the context of cancer treatment (Figure 1(b)). Since a tremendous interest in acupuncture and herbal medicine was observed in 2000, the increasing numbers of published papers per year are apparent. In 2013, more than 90 papers on acupuncture and more than 450 papers on herbal medicines appeared in the area of cancer prevention and therapy. The increase rate of published papers concerning cancer over the years indicates that this small research field took a booming development. These facts and figures illustrate that the present special issue focuses on a hot topic in cancer research.

For this year's special issue, we were able to gather a panel of scientists from different fields working on innovative topics. The papers of this special issue include the following: “*The effects of herbs and fruits on leukemia*” by T. A. Saedi et al., “*Medicinal plants and other living organisms with antitumor potential against lung cancer*” by L. S. Monteiro et al., “*Pharmacopuncture for cancer care: a systemic review*” by S. Cheon et al., “*Acupuncture for preventing complications after radical hysterectomy: a randomized controlled clinical trial*” by W. Yi et al., “*Antitumor activity of Chinese propolis in human breast cancer MCF-7 and MDA-MB-231 cells*” by H. Xuan et al., and “*Apoptosis induction by the total flavonoids from Arachnoides exilis in HepG2 cells through reactive oxygen species-mediated mitochondrial dysfunction involving MAPK activation*” by H. Li et al.

We, the editors, believe this issue may be of particular interest to the readers. Hopefully, this issue will give helpful insights to researchers, physicians, and patients and will also be able to make a progress in the field of cancer CAM.

*Thomas Efferth* *Thomas Efferth* *Sookyung Lee* *Sookyung Lee* *Yoshiharu Motoo* *Yoshiharu Motoo* *Sven Schröder* *Sven Schröder*

## Figures and Tables

**Figure 1:**
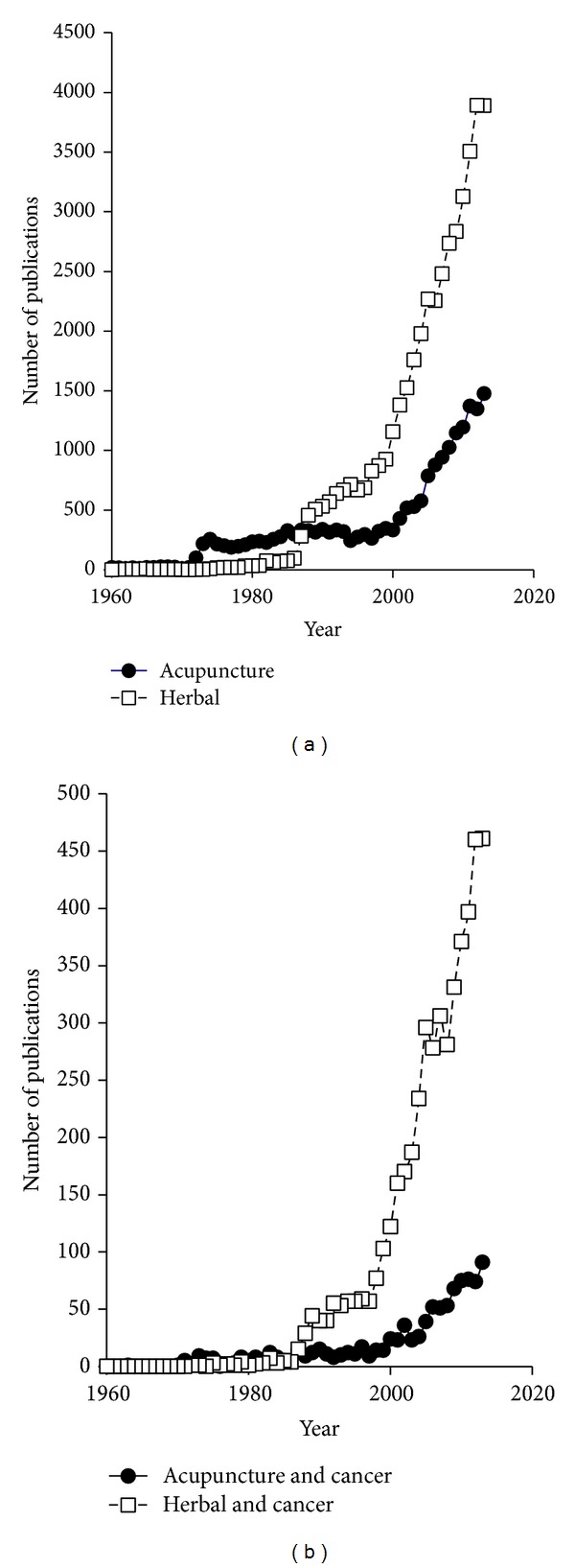
Survey of literature deposited in the PubMed database from 1960 to 2013 with the keywords (a) “acupuncture” or “herbal” and (b) “acupuncture and cancer” or “herbal and cancer.”

